# Long-Term Follow-Up of the Fellow Eye in Patients Undergoing Surgery on One Eye for Treating Myopic Traction Maculopathy

**DOI:** 10.1155/2016/2989086

**Published:** 2016-07-12

**Authors:** Hui-Juan Xia, Wei-Jun Wang, Feng'E Chen, Ying Wu, Zhen-Yuan Cai, Wei Chen, Su-Qin Yu, Ying Fan

**Affiliations:** The First People's Hospital, Shanghai Jiaotong University, Shanghai 200080, China

## Abstract

*Objective*. To observe the fellow eye in patients undergoing surgery on one eye for treating myopic traction maculopathy.* Methods*. 99 fellow eyes of consecutive patients who underwent unilateral surgery to treat MTM were retrospectively evaluated. All patients underwent thorough ophthalmologic examinations, including age, gender, duration of follow-up, refraction, axial length, intraocular pressure, lens status, presence/absence of a staphyloma, and best-corrected visual acuity (BCVA). Fundus photographs and SD-OCT images were obtained. When feasible, MP-1 microperimetry was performed to evaluate macular sensitivity and fixation stability.* Results*. At an average follow-up time of 24.7 months, 7% fellow eyes exhibited partial or complete MTM resolution, 68% stabilized, and 25% exhibited progression of MTM. Of the 38 eyes with “normal” macular structure on initial examination, 11% exhibited disease progression. The difference in progression rates in Groups 2, 3, and 4 was statistically significant. Refraction, axial length, the frequency of a posterior staphyloma, chorioretinal atrophy, initial BCVA, final BCVA, and retinal sensitivity all differed significantly among Groups 1–4.* Conclusions*. Long axial length, chorioretinal atrophy, a posterior staphyloma, and anterior traction contribute to MTM development. Patients with high myopia and unilateral MTM require regular OCT monitoring of the fellow eye to assess progression to myopic pre-MTM. For cases exhibiting one or more potential risk factors, early surgical intervention may maximize the visual outcomes.

## 1. Introduction

High myopia is associated with a lens diopter ≤ −6 D and an axial length ≥ 26 mm. Such a condition is also termed malignant or pathologic myopia when accompanied by choroid retinal degeneration and a series of pathological changes in the macular region [1]. High myopia is very common, found in 9–21% [2] of Asian adults, and its incidence is increasing worldwide. With the advent of optical coherence tomography (OCT), abnormal structures in the posterior pole of the retina are now readily detectable and various OCT-driven treatments have been developed. Panozzo and Mercanti [3] proposed unifying all pathological features generated by traction in the context of myopia under a condition termed “myopic traction maculopathy” (MTM). This is a form of degenerative disease reported in 9–34% of highly myopic eyes with posterior staphylomas [3, 4]. The various clinical manifestations impact patient vision and function, eventually affecting the quality of life.

Neither the pathogenesis nor the triggers of MTM development are well understood. Tangential traction of the vitreous cortex, development of an epiretinal membrane, inflexibility of retinal vessels, and development of a posterior staphyloma are all thought to play a role [5]. There are few reports pertaining to the natural history of MTM and treatment responses to this condition. Indeed, much debate remains regarding whether treatments seeking to reduce MTM are appropriate and, if so, which treatment is optimal. The visual acuity required for reading usually remains compromised for an extended period of time after surgery; therefore, prophylactic measures for the fellow eye should be considered. Few long-term follow-up studies on the fellow eyes of patients undergoing MTM surgery have been published. We thus explored the fellow eyes of such patients, describing the visual changes noted in several cases undergoing spontaneous resolution and the potential mechanisms involved.

## 2. Materials and Methods

Ninety-nine consecutive patients underwent unilateral surgery to treat MTM at the First People's Hospital affiliated to Shanghai Jiaotong University. The Shanghai First People's Hospital Ethics Committee provided written approval for the study. All participants provided written informed consent. The fellow eyes were retrospectively evaluated.

All patients underwent thorough ophthalmologic examinations. We recorded age, gender, duration of follow-up, refraction, axial length, intraocular pressure, lens status, presence/absence of a staphyloma, and best-corrected visual acuity (BCVA). Fundus photographs and SD-OCT images (Spectralis, Heidelberg Engineering, Germany) were obtained. When feasible, MP-1 microperimetry (Padua, Nidek Technologies, Italy) was performed to evaluate macular sensitivity and fixation stability. B-scan ultrasonography was used to confirm posterior staphyloma. Axial length was measured by IOL Master (Zeiss, Germany). Both the initial and final BCVA values were expressed in LogMAR units.

Patients were divided into five groups according to baseline macular structure on OCT [6, 7]: (1) Group 1 (*n* = 38) “normal”: the macula was normal in terms of structure and thickness, with no evident change in the foveal contour of the underlying retinal tissues; (2) Group 2 (*n* = 42) “macular retinoschisis”: presence of macular distortion (e.g., retinal thickening, a lamellar macular hole, and macular retinoschisis), often associated with vitreomacular traction or the presence of an epiretinal membrane; (3) Group 3 (*n* = 10) “foveal detachment”: presence of photoreceptors detachment from the retinal pigment epithelium in the region of the fovea, with or without retinoschisis; (4) Group 4 (*n* = 5) “full-thickness macular hole”: presence of interruption of all retinal layers from the internal limiting membrane to the retinal pigment epithelium, with or without retinal detachment; (5) Group 5 (*n* = 4) “other”: macular degeneration group featuring macular atrophy and neovascularization.

Statistical analysis was performed using commercial software (SPSS version 19.0; SPSS Inc., Chicago, IL, United States). Continuous values were expressed as means ± standard deviation (SD) or as medians. The significance of differences among continuous variables was assessed using the Kruskal-Wallis test. The significance of differences among noncontinuous variables was analyzed using *χ* ^2^ and Fisher exact tests. Significance was set at *P* < 0.05.

## 3. Results

Baseline demographic and clinical information on the 99 patients are summarized in Tables 1 and 2. A posterior staphyloma was evident in 72 of 99 eyes (73%). Diffuse or patchy atrophy was observed in 43 eyes. Sixty-one patients (62%) exhibited pathological changes in both eyes on initial examination. At an average follow-up time of 24.7 months, 7 out of 99 fellow eyes (7%) exhibited partial or complete MTM resolution and 3 were from spontaneous release of retinal traction evident on OCT. Of all followed up eyes, 67 out of 99 eyes (68%) stabilized and 25 (25%) exhibited progression of myopic macular maculopathy. Thirteen eyes required vitrectomy for clinically significant vision decrease and/or metamorphopsia.

Of the 38 eyes with “normal” macular structure on initial examination, 11% exhibited disease progression. The progression rates in Groups 2, 3, and 4 were 24%, 60%, and 60%, respectively. The difference was statistically significant (Fisher exact probability test, *P* = 0.005). Refraction, axial length, the frequency of a posterior staphyloma, chorioretinal atrophy, initial BCVA, final BCVA, and retinal sensitivity all differed significantly among Groups 1–4 (*P* = 0.015, 0.016, 0.004, < 0.001, < 0.001, < 0.001, and 0.003, resp.).

Separation caused by anterior retinal traction was detected by OCT in three of the seven cases exhibiting spontaneous resolution or improvement. We next describe the remaining four cases. They were of great interest in terms of the mechanisms of spontaneous resolution (which have been very rarely studied) and the visual outcomes achieved.

**Figure 1:**
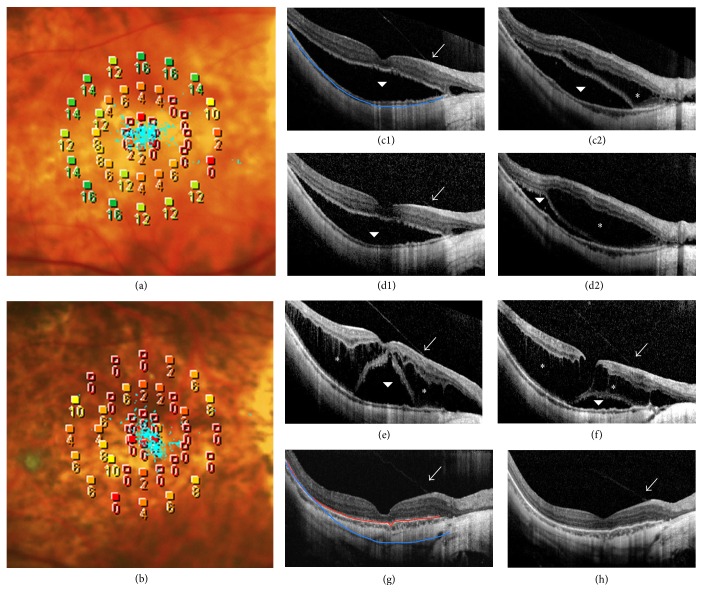
OCT and MP changes in Case 1 during follow-up. (a) Microperimetric image shows that retinal sensitivity was 6.85 dB and fixation was stable at baseline. (b) After 66 months, retinal sensitivity was 2.45 dB and fixation remained stable. (c1) Baseline OCT through the fovea reveals foveal detachment (arrowhead) and an attachment of the vitreous (arrow) apparent at the retinal surface. (c2) Baseline OCT taken inferior to the macula reveals macular retinoschisis (asterisk) and foveal detachment (arrowhead). (d1)-(d2) After 2 months, OCT reveals mild improvement in the foveal detachment but with retinoschisis progression. (e)–(h) OCT through the fovea acquired at 11, 33, 64, and 66 months, respectively.

**Figure 2:**
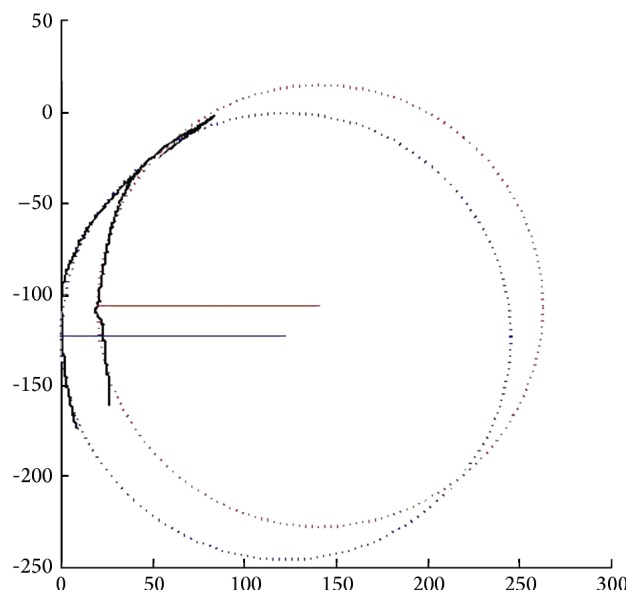
Method of curvature calculation. The curvatures of the extracted traces were calculated in Matlab (R2014 a, Mathworks). A circle in Cartesian coordinate was fit to each trace with outputs of the center point and radius. The fitted circles were plotted and overlaid with the original traces. R (blue) = 123.0231; R (red) = 121.7396.

*Case 1*. A 66-year-old female with high myopia complained of decreased vision in her right eye (OD). On initial examination, the BCVA was 20/133 and the retinal sensitivity was 6.85 dB with stable fixation OD (Figure 1(a)). OCT revealed foveal detachment with partial posterior vitreous detachment (the vitreous remained attached at the parafoveal area, Figures 1(c1) and 1(c2)). Two months later, OCT revealed a mild decrease in retinal thickness and the presence of subfoveal fluid. Retinoschisis was apparent inferior to the macula (Figures 1(d1) and 1(d2)). At 11 months, the retina was largely reattached but the intralayer retinoschisis had increased (Figure 1(e)). Vitreous traction was still evident (with no change on OCT). At 33 months, both the foveal detachment and the retinoschisis had decreased (Figure 1(f)). At the final visit (66 months after initial presentation), the retinal structure was reattached but with a discontinuous band in the photoreceptor layer (Figure 1(h)). And the posterior scleral curvature on final OCT images was flatter than that on initial visit, with its radius changing from 123.0231 (blue line) to 121.7396 (red line) (Figure 2). The BCVA remained at 20/200 and retinal sensitivity was 2.45 Db with stable fixation on final examination. On her last follow-up visit, vision had not yet improved.

**Figure 3:**
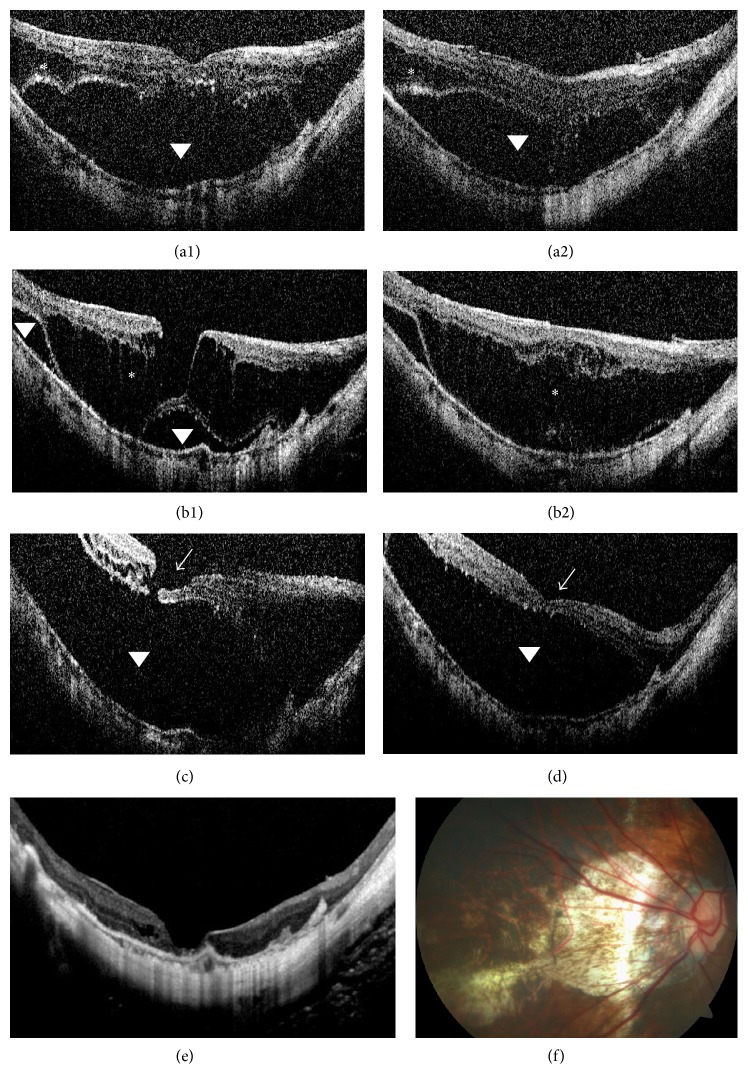
OCT findings in Case 2 during follow-up. (a1) Baseline OCT through the fovea reveals macular retinoschisis (asterisk) and foveal detachment (arrowhead). (a2) Baseline OCT taken inferior to the macula at baseline. (b1)-(b2) After 11 months, OCT reveals retinoschisis and a lamellar hole, accompanied by reduced foveal detachment. (c) After 25 months, OCT reveals that the foveal detachment has recurred and a small macular hole (arrowhead) has formed. (d) After 35 months, OCT reveals that retinal thickness has decreased and retinoschisis has improved. (e) After 65 months, OCT reveals retinal reattachment and a macular hole. (f) At 65 months, fundus photograph depicts patchy retinochoroidal atrophy in the posterior pole.

*Case 2*. A 64-year-old female with high myopia initially presented for consideration of binocular cataract surgery. Initial OCT revealed OD foveal detachment and retinoschisis (Figures 3(a1) and 3(a2)). After phacoemulsification and lens implantation, BCVA was 20/80. Eleven months later, retinoschisis was evident, as was a lamellar hole, with improved foveal detachment (Figures 3(b1) and 3(b2)). However, such detachment recurred and a small macular hole developed over the next 14 months of follow-up (Figure 3(c)), accompanied by BCVA deterioration from 20/80 to 20/200. At 35 months, OCT revealed incomplete spontaneous resolution of the foveal detachment, the retinoschisis, and the retinal thickness (Figure 3(d)). No PVD was evident, but a membrane adherent to the parafovea persisted during long-term follow-up. The photoreceptor layer disappeared, likely due to long-term detachment from the retinal pigment epithelium layer, and BCVA was at the counting fingers level. The last OCT examination performed 65 months postoperatively revealed retinal reattachment and a macular hole (Figure 3(e)). Fundus photography revealed patchy retinochoroidal atrophy in the posterior pole (Figure 3(f)). The BCVA in the right eye remained at the counting fingers level.

**Figure 4:**
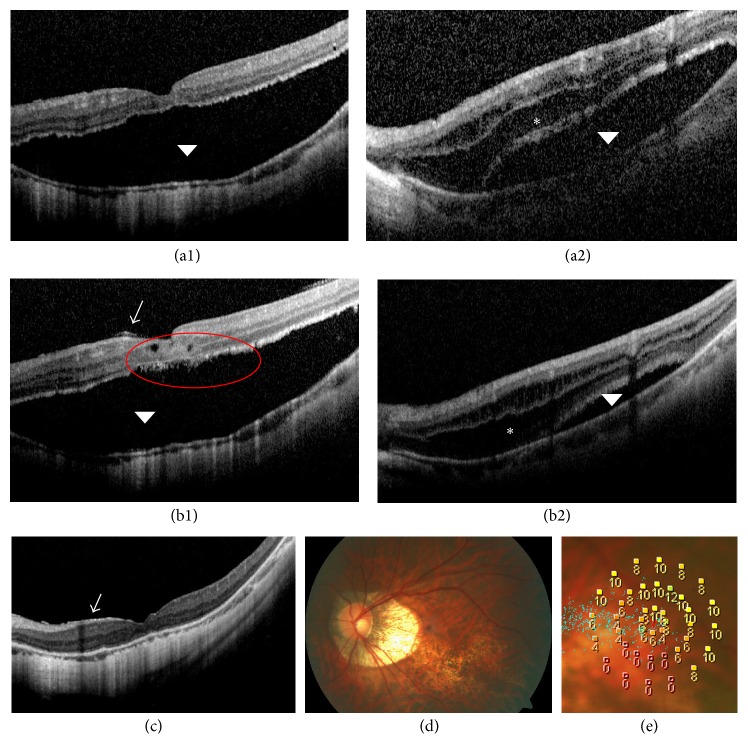
OCT findings in Case 3 during follow-up. (a1) Baseline OCT of the left eye (OS) reveals foveal detachment (arrowhead). (a2) Baseline OS OCT taken superior to the fovea reveals macular retinoschisis (asterisk) and foveal detachment (arrowhead). (b1)-(b2) OS OCT 1 month later reveals that the foveal detachment has reduced, photoreceptor layer is discontinuous (red circle), and retinoschisis has increased in the region superior to the fovea. A thin flat membrane (arrow) is evident. (c) At 44 months, OS OCT reveals that retinal traction has spontaneously resolved. (d) OS fundus photograph taken 44 months later depicts some atrophy in the inferior macula. (e) At 44 months, OS microperimetric image reveals that the retinal sensitivity was 6.30 dB and that fixation was unstable.

*Case 3*. A 55-year-old male with high myopia was referred for binocular vision loss. OCT revealed an OD macular hole with retinal detachment and foveal detachment in the left eye (OS, Figures 4(a1) and 4(a2)). BCVA was counting fingers OD and 20/200 OS. OD pars plana vitrectomy, membrane peel, and gas were performed and close follow-up was scheduled for the left eye. One month later, OCT revealed reduced foveal detachment and a discontinuous photoreceptor layer; the outer layer had begun to attach to the retinal pigment epithelial layer and retinoschisis had developed superior to the fovea (Figures 4(b1) and 4(b2)). At 44 months, the lesion had spontaneously resolved but the photoreceptor layer was absent (Figures 4(c)–4(e)). A thin flat membrane persisted around the fovea. This condition remained unchanged for the ensuing 9 months. The final BCVA was 20/200 and the macular sensitivity was 6.30 dB with unstable fixation on microperimetry. Unfortunately, the patient continued with a subjective complaint of a central fixed shadow which persisted throughout his long-term follow-up.

**Figure 5:**
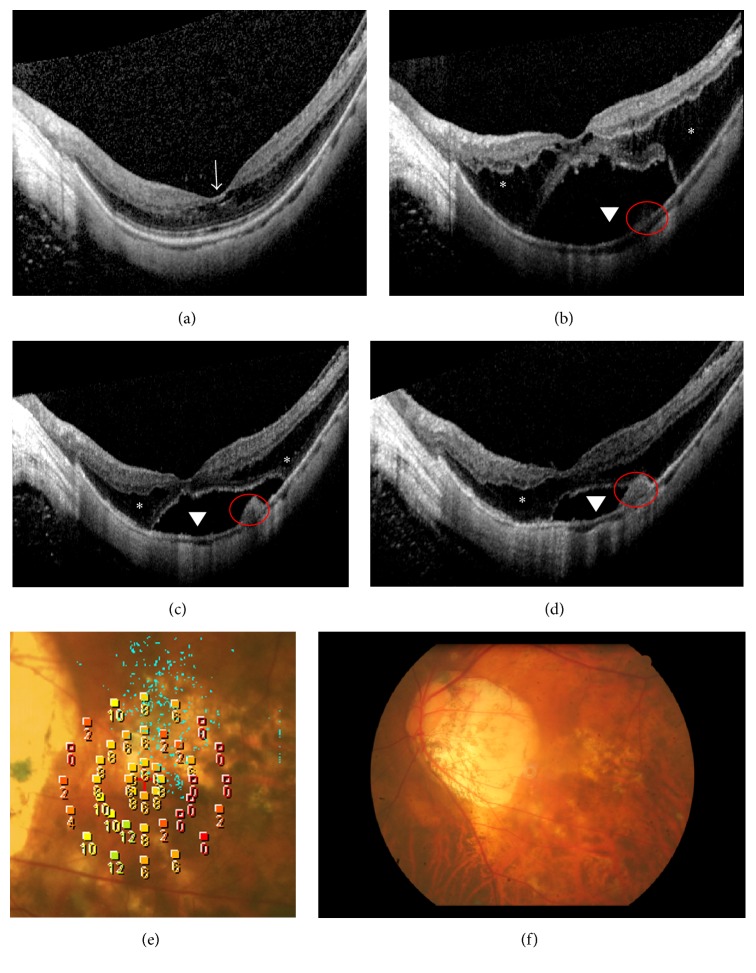
OCT findings in Case 4 during follow-up. (a) Baseline OCT reveals mild macular retinoschisis (arrow). (b) At 19 months, OCT reveals progression of myopic maculopathy with foveal detachment (arrowhead), retinoschisis (asterisk), and CNV (red circle) evident. (c) At 28 months, OCT reveals improvement in retinoschisis and foveal detachment. (d) At 39 months, OCT reveals further improvement. (e) At 39 months, microperimetric image reveals that retinal sensitivity was 5.10 dB and with unstable fixation. (f) At 39 months, fundus photograph depicts some atrophy in the central macula.

*Case 4*. A 64-year-old female with high myopia underwent successful surgical repair of OD foveal detachment. The left eye was treated conservatively for minimal foveoschisis and epiretinal membrane evident on initial OCT (Figure 5(a)). BCVA was 20/67. Nineteen months later, OCT revealed progression of myopic maculopathy (Figure 5(b)) evidenced by increased thickness and extension of retinoschisis, with an apparent foveal detachment. A small, highly reflective dome-like elevation of the retinal pigment epithelium, reaching the parafovea, was present, suggestive of myopic choroidal neovascularization (CNV). BCVA had fallen to 20/100. At 28 months, OCT revealed improvements in terms of both retinoschisis and foveal detachment (Figure 5(c)). At 39 months (Figures 5(d)–5(f)), there was further improvement on OCT. The CNV-like highly reflective elevation persisted. BCVA remained at 20/100 and microperimetry demonstrated that macular sensitivity was 5.10 dB with unstable fixation. The epiretinal membrane remained unchanged throughout follow-up.

## 4. Discussion

The prevalence of MTM is 9–34% [3, 4] in highly myopic eyes with posterior staphylomas. MTM can present with vitreoretinal traction, schisis-like thickening, a lamellar or full-thickness macular hole, foveal detachment, or a macular hole associated with retinal detachment [5]. Given the range of MTM manifestations and pathogenesis, the resulting retinal impairment is also variable and surgical timing and choice of intervention remain controversial.

In the present study, 57 patients (57.6%) exhibited fellow eye MTM on initial examination after surgical repair of MTM on the contralateral eye; this prevalence was thus greater than that reported previously. Shimada et al. [8] reported that 11.6% of high myopic eyes exhibited MTM progression. The prevalence of MTM progression was 23.2% in the fellow eyes in the present study over an average follow-up of 24.7 months (range of 6 to 64 months). These differences in prevalence and the extent of progression may be attributable to subject selection. In other words, a patient with MTM in one eye may be more likely to have MTM in the other eye. Similarly, Tsujikawa et al. observed that high myopic patients, in whom one eye develops a macular hole with retinal detachment, would be expected to be at an increased risk of retinal detachment in the fellow eye [9].

Severe macular maculopathy evident on baseline OCT images of fellow eyes was notably progressive during follow-up, especially in eyes exhibiting extensive foveal traction or clinical significant posterior staphylomas. The risk of deterioration of Group 2 eyes with macular retinoschisis was lower than that of eyes exhibiting foveal detachment and/or a macular hole but remained higher than that of the normal group. Thus, the more severe the MTM on baseline exam, the more progressive the condition. Gaucher et al. [10] showed that myopic foveoschisis with a change in premacular structure seemed to develop more frequently in patients exhibiting foveal detachment with or without a macular hole, with progression evident in 20 of 29 eyes. Several studies [10–12] have found that foveal detachment and/or abnormal traction in the anterior retina were risk factors for MTM progression and could trigger rapid loss of vision. Theodossiadis et al. [13, 14] suggested that preservation of meaningful residual vision necessitates that the photoreceptor and external limiting membrane layers remain intact. We found that outer retinal lesions were common in MTM eyes, especially those of Groups 3 and 4. In patients undergoing frequent OCT, the status of the photoreceptor layer (intact, disrupted, or disappeared) was carefully noted during long-term follow-up.

We also found that refraction, axial length, posterior staphyloma status, and the extent of choroidoretinal atrophy differed significantly among the four subgroups. A few reports [4, 15–18] found that axial length, atrophy, and a posterior staphyloma were associated with an increased risk of MTM in high myopic eyes and influenced the natural course of MTM. Large-scale studies featuring multiple regression analyses are needed to determine whether these variables independently predict MTM development.

The evolution of MTM is variable. We found that 67.7% of fellow eyes were stable after MTM surgery, whereas 23.2% deteriorated during long-term follow-up. Ikuno et al. [7] found that the natural progression of macular retinoschisis was from initial retinoschisis to foveal detachment and finally to a macular hole with retinal detachment. We found that several of our progressive cases followed certain patterns, but in most cases there was minimal progression. Even longer-term follow-up may be required to observe these cases. Within the extent of our follow-up, 7 cases fortunately underwent spontaneous reduction or complete resolution of MTM. Since the pathology of MTM remains largely unelucidated, as the mechanisms of spontaneous resolution. the associated changes in visual function require further research.

Of the seven cases exhibiting spontaneous resolution, elimination of anterior retinal traction was evident in three, resulting in anatomic restoration of the retina. Shimada et al. [8] reported that 8 of 207 MTM eyes exhibited partial or complete resolution after posterior vitreous detachment or spontaneous internal limiting membrane disruption, during a mean follow-up time of 36.2 months. Hirota et al. [19] described four instances of spontaneous resolution in MTM eyes in which the vitreofoveal anterior traction was released. This is in line with the fact that vitrectomy effectively eliminates abnormal vitreoretinal traction. The pharmacological agents used to treat persistent pathological vitreomacular adhesions are also of great interest [20, 21].

In the other four cases we described above, we observed persistent vitreoretinal adhesion over the macula, which appeared to remain unchanged on OCT during long-term follow-up. This is consistent with the suggestion by Goldman and Duker [22] that a spontaneous improvement in the macular contour may be attributable to spontaneous release of an anterior vitreous adhesion lying outside of the imaging field. Some rare cases exhibit progressive development of damage after the vitreoretinal interface traction is released. This suggests that vitreoretinal traction over the macula is not the only (or not the major) pathophysiological mechanism in play; therefore, further research is required. Furthermore, the images of Case 2 revealed retinal reattachment, accompanied by formation of a macular hole. This may be due to tissue dehiscence as an adaptive response in order to release the tractional force exerted from both the vitreoretinal interface anteriorly and the staphyloma posteriorly that results in the retinal thickening in retinoschisis. If the resulting macular hole is small, subretinal fluid would theoretically be absorbed by the retinal pigment epithelium pump, restoring the retinal anatomy. Li et al. [23] and Yu et al. [24] found that even a myopic macular hole with retinal detachment could resolve spontaneously without release of vitreoretinal traction; however, the mechanism remained unclear.

Curtin divided posterior staphylomas into ten types and concluded that the posterior pole (Type I) and macular (Type II) staphylomas were the most common [25]. A posterior staphyloma in a high myopic eye has been shown not only to deepen with age but also to change in shape over time [26]. We can see the posterior scleral curvature on final OCT images was flatter than that on initial visit in Case 1. We thus postulate that an anatomical or structural change within the sclera can flatten a posterior scleral, eventually restoring the retina. This may imply that posterior scleral shape plays a role in the progression. In recent years, the research on dome-shaped macula demonstrated that the bulge in eyes with a dome-shaped macula may act as a macular buckle-like mechanism, indenting the fovea similar to a macular exoplant or Ando plomb device, and thus may prevent or alleviate tractional forces over the fovea, thereby preventing retinoschisis or detachment [27]. Although findings in this case were different from a typical dome-shaped macula, the mechanism they acted may be similar. Although this requires confirmation and needs further objective measures of tracking posterior scleral curvature, it is interesting to note that posterior scleral buckling might be a valuable alternative or adjunctive treatment for such a condition.

Notably, the outer retina, not the entire retina, began to adhere to the retinal pigment epithelium with retinoschisis progression. We therefore hypothesize that, in addition to the (probable) partial release of anterior traction, factors external to the retina are also involved. Liquid accumulation in the retina, caused by disturbance of choroid retinal vascular fluid flow or inflammation, may also be involved. As inflammation lessens, the lesion would theoretically improve. MTM and CNV coexisted in Case 4, and it is unclear whether the CNV influenced foveal detachment or the course of spontaneous improvement.

Visual function after resolution is important. Although anatomical resolution was apparent, vision did not improve in our four cases. Recently, it has become clear that visual function requires outer retinal integrity [28–30]. Photoreceptor cells are irreversibly impaired when they are detached from the retinal pigment epithelial layer for a long time, seriously compromising visual functional recovery [31].

Our study is limited by its retrospective nature. Currently, no consensus on MTM classification (which would facilitate further research) has been attained. Not all fellow eyes exhibited high myopia; some were even emmetropic (10/99). MTM pathology and development may differ in such eyes, thereby biasing our results.

## 5. Conclusion

We engaged in long-term follow-up of the fellow eyes of eyes in patients that previously underwent unilateral surgical repair of MTM and found these fellow eyes were at an increased risk of MTM development and progression. We suggest that a long axial length, chorioretinal atrophy, a posterior staphyloma, and anterior traction contribute to MTM development. MTM may rarely improve naturally and we describe a few such cases. Patients with high myopia and unilateral MTM require regular OCT monitoring of the fellow eye to assess progression to myopic pre-MTM. For cases exhibiting one or more potential risk factors, early surgical intervention may maximize the visual outcomes.

## Figures and Tables

**Table 1:** Summary of subject demographic and clinical characteristics.

	*n* = 99
Sex (#)	
Male	18
Female	81

Age, years	
Mean ± SD	62.57 ± 7.94
Range	44–85

Axial length, mm	
Mean ± SD	29.10 ± 2.37
Range	23.07–36.09

Refraction, diopters	
Mean ± SD	−12.59 ± 5.43
Range	−23.00–0.00

Follow-up, months	
Mean ± SD	24.73 ± 16.85
Range	6–64

Lens status (#)	
Phakic	83
IOL	16

LogMAR BCVA	
Baseline (median)	0.3
Final (median)	0.4

Final retinal sensitivity, dB	
Mean ± SD	8.80 ± 4.68
Range	0–15.65

Posterior staphyloma (#)	72

Choroidoretinal atrophy (#)	43

#: number. BCVA: best-corrected visual acuity. dB: decibels.

IOL: intraocular lens. mm: millimeters. SD: standard deviation.

**Table 2:** Fellow eye characteristics in patients with MTM surgery.

	Group 1 (*n* = 38)	Group 2 (*n* = 42)	Group 3 (*n* = 10)	Group 4 (*n* = 5)	*P* value
Age, years	63.26 ± 8.29	61.40 ± 6.91	62.50 ± 6.52	66.00 ± 10.93	0.664^a^

Sex (male/female)	8/30	7/35	2/8	1/4	0.970^b^

Refraction, D	−10.21 ± 6.15	−13.89 ± 4.50	−14.63 ± 3.74	−14.00 ± 4.74	0.015^a^

Axial length, mm	28.21 ± 2.78	29.53 ± 1.99	30.20 ± 1.24	30.04 ± 2.41	0.016^a^

Follow-up, months	22.55 ± 17.54	23.12 ± 14.32	34.80 ± 20.86	34.60 ± 17.16	0.138^a^

Poststaphyloma, number					
Yes	20	34	10	4	0.004^b^
No	18	8	0	1	

Chorioretinal atrophy					
Yes	7	19	9	4	<0.001^b^
No	31	23	1	1	

OCT change, number					
Improved	0	4	3	0	0.005^b#^
Stable	34	28	1	2	
Worse	4	10	6	3	

BCVA^*※*^					
Baseline	0.10	0.40	1.0	0.9	<0.001^a^
Final	0.20	0.45	1.0	0.9	<0.001^a^

RS, dB (final)	10.24 ± 4.10	8.83 ± 4.53	4.87 ± 4.03	5.79 ± 6.02	0.003^a^

^a^Kruskal-Wallis test. ^b^Fisher's exact test. ^#^compared with Groups 2, 3, and 4. ^*※*^Median.

BCVA: best-corrected visual acuity. RS: retinal sensitivity.
